# Assessing Acetabular Index Angle in Infants: A Deep Learning-Based Novel Approach

**DOI:** 10.3390/jimaging9110242

**Published:** 2023-11-06

**Authors:** Farmanullah Jan, Atta Rahman, Roaa Busaleh, Haya Alwarthan, Samar Aljaser, Sukainah Al-Towailib, Safiyah Alshammari, Khadeejah Rasheed Alhindi, Asrar Almogbil, Dalal A. Bubshait, Mohammed Imran Basheer Ahmed

**Affiliations:** 1 Department of Computer Science, College of Computer Science and Information Technology, Imam Abdulrahman Bin Faisal University, P.O. Box 1982, Dammam 31441, Saudi Arabiakralhindi@iau.edu.sa (K.R.A.);; 2 Department of Orthopedics, College of Medicine, Imam Abdulrahman Bin Faisal University, P.O. Box 1982, Dammam 31441, Saudi Arabia; 3 Department of Computer Engineering, College of Computer Science and Information Technology, Imam Abdulrahman Bin Faisal University, P.O. Box 1982, Dammam 31441, Saudi Arabia

**Keywords:** development dysplasia of the hip (DDH), deep learning, acetabular index, Mask-RCNN, key point, RCNN, Detectron2

## Abstract

Developmental dysplasia of the hip (DDH) is a disorder characterized by abnormal hip development that frequently manifests in infancy and early childhood. Preventing DDH from occurring relies on a timely and accurate diagnosis, which requires careful assessment by medical specialists during early X-ray scans. However, this process can be challenging for medical personnel to achieve without proper training. To address this challenge, we propose a computational framework to detect DDH in pelvic X-ray imaging of infants that utilizes a pipelined deep learning-based technique consisting of two stages: instance segmentation and keypoint detection models to measure acetabular index angle and assess DDH affliction in the presented case. The main aim of this process is to provide an objective and unified approach to DDH diagnosis. The model achieved an average pixel error of 2.862 ± 2.392 and an error range of 2.402 ± 1.963° for the acetabular angle measurement relative to the ground truth annotation. Ultimately, the deep-learning model will be integrated into the fully developed mobile application to make it easily accessible for medical specialists to test and evaluate. This will reduce the burden on medical specialists while providing an accurate and explainable DDH diagnosis for infants, thereby increasing their chances of successful treatment and recovery.

## 1. Introduction

Developmental dysplasia of the hip (DDH) is the most common musculoskeletal developmental disorder that affects newborns and is regarded as the primary cause of around 25% to 43% of end-stage hip arthroplasty cases [1,2]. DDH poses a significant risk to the quality of life for patients from an early age and could persist into adulthood without proper treatment [3]. Failure to recognize and diagnose DDH in its early stages can lead to functional impairment, chronic hip pain, progressive hip degeneration, and accelerated osteoarthritis that necessitates surgical intervention later in life [4].

DDH occurs when the femoral head does not fit into the socket due to an abnormal hip joint, leading to limited hip range of motion, and noticeable gait abnormalities. Early identification through reliable hip diagnostic screening is crucial for timely intervention [5]. Detecting DDH at an early age significantly reduces risks of long-term complications.

**Figure 1:**
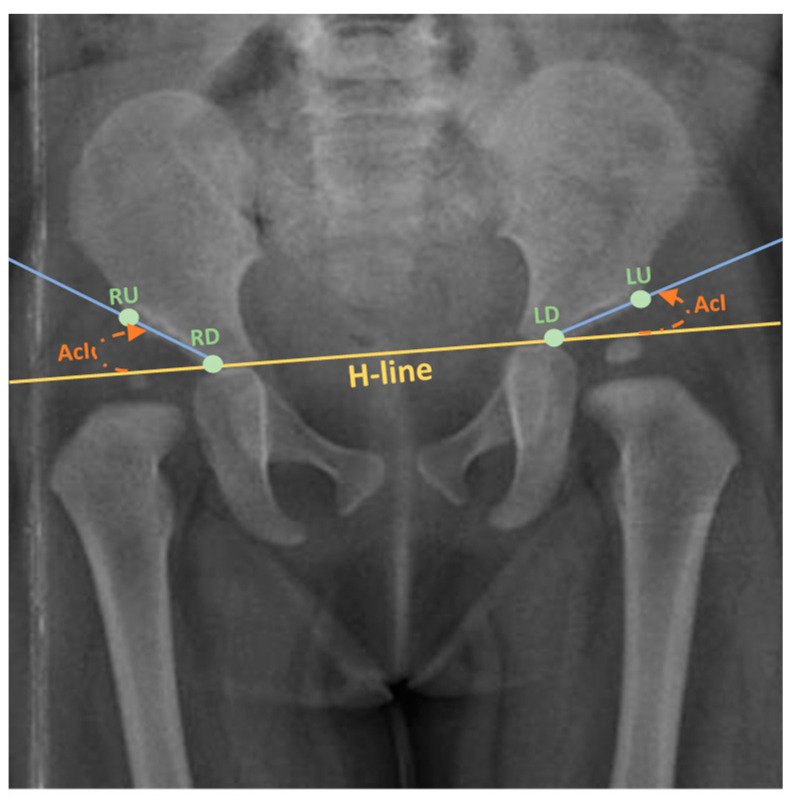
Calculating the AcI, with H-line displayed (Adapted from [7]). Keypoints: LU: Left upper; LD: Left down; RU: Right upper; RD: Right down.

The current clinical approach for diagnosing DDH involves manual measurements of various anatomical characteristics from pelvic radiographs, followed by assessment by experienced clinicians or radiologists. However, this process lacks standardization and heavily relies on subjective metrics, demanding a greater degree of knowledge and clinical experience. Recent advancement in computer-aided medical diagnosis (CAD) supplemented with pattern recognition techniques have made it feasible to incorporate these technologies into practical solutions that offer an automated, standardized, and reliable diagnosis of DDH in infants. These solutions utilize the acetabular index angle measurement (AcI) as a clinically established radiographic measurement for assessing the severity of DDH in individual cases (Figure 1). AcI is measured by calculating the angle between two lines. The first line extends from the medial edge to the most lateral aspect of the acetabular sourcil, which refers to the inner rim of the acetabulum. The second line, known as Hilgenreiner’s line, or H-line, runs horizontally on the pelvis. The degree of the angle formed by their intersection indicates the range within which the acetabular index falls. An increased acetabular inclination range is indicative of the presence of DDH [6].

The objective of this study is to present and investigate a pipelined deep-learning framework for diagnosing DDH. The proposed framework leverages Detectron2 Mask RCNN for accurate image segmentation. Subsequently, the segmented images are processed through a keypoint detection network to identify the relevant landmarks required for calculating the AcI and assessing the severity of DDH. It is noteworthy that DDH occurrence cannot be prevented, though an early diagnostic can help decrease the complications. Moreover, mass screening is difficult since not everyone can read or understand it. To the best of our knowledge, there is a need for an automated software tool for assessing DDH in clinical practice. Hence, the implementation of the proposed framework in a fully-fledged mobile application should be of great use to hospitals worldwide.

### 1.1. Related Literature

There have already been several advances in utilizing CAD to assess DDH in the last decades. The first attempt to automatically detect relevant landmarks from medical images of the infant pelvis could be traced back to 1997, when Overhoff et al. [8], introduced an image processing-based approach to determine the region of interest (ROI) of the acetabulum and approximate the sphere of the femoral head in 3D ultrasound images. The findings of the study were further extended [9] to detect the acetabular landmarks used to assess the relevant angle measurements. More image processing-based algorithms such as the Hough transform were proposed [10] to offer a fully automated approach for the measurement of the acetabular cartilage in MRI medical images. Moreover, in a more recent study, Hough transform was paired with canny edge detection to assess relevant landmarks on the pelvic X-ray images [11].

#### 1.1.1. Detection & Diagnosis Software Tools

Many medical professionals use CAD-based tools to make assessments in clinical settings for DDH diagnosis and AcI quick measurement. Acetabular Index, an application for portable devices, calculates the index via manually detected landmarks. It measures angles in X-ray images through the aid of a circular transparent template, where the points of interest are marked accurately. The lines automatically formed between points serve to measure the angles of interest. Although an abundance of research attempts to propose a reliable method for automating medical diagnosis, few are deployed and utilized in professional environments. BoneView is a good representation of a fully-fledged medical software powered by AI that offers diagnostic features for bone lesions, fractures, and dislocations detection for bone trauma X-rays. Techniques used in BoneView include a ‘Detectron 2’-based diffusion-convolutional neural network (DCNN) model developed for image training. In addition, it applies natural language processing (NLP) algorithms to infer diagnostic interpretations from radiologists’ reports [12]. Similarly, ImageBiopsy Lab launched several software products to detect anomalies in the musculoskeletal system. One product is IB Lab LAMA for automating the assessment of pre-and post-total knee arthroplasty (TKA) lower limb alignment using AI methodologies in addition to “HIPPO” for hip angles and morphology assessments [13,14,15,16,17].

#### 1.1.2. Research Utilizing ML/DL-Based Methodologies

Although traditional image processing-based CAD approaches have achieved acceptable results, they struggle upon implementation with larger and more diverse datasets. In addition, they have a lengthy processing procedure that is impractical in medical environments. That is where artificial intelligence (AI) concepts such as machine learning (ML) and deep learning (DL) come into the picture with a variety of algorithms and frameworks that had been implemented for several purposes such as classification, object detection, instance segmentation, and keypoint detection. Several ML- and DL-based solutions have been utilized in anatomical landmark detection and segmentation in the medical X-ray imaging of the pelvis. Thompson et al. [18] utilized a random-forest-based method for measuring the radiograph’s angular parameters (AcI, RMP) based on automatically detected anatomical landmarks. The proposed system was tested and validated on a clinical dataset of 200 cases. Another ML model was proposed by Jiang et al. [19] using a computer-aided system (CAD) to semi-automatically measure the Tönnis angle, Sharp angle, and CE angle following the bone contours of the hip joint. The CAD system consists of four stages that involve manual bone outlining, automatic landmarks extraction, automatic angle measurement, and hip development assessment and classification using a machine-learning modal support vector machine (SVM). The dataset consisted of 248 X-ray images from Zhongshan Hospital of Dalian University, China.

Using a dataset of 9369 images for patients aged from 0.1 to 14 years, Jingyuan Xu et al. [20] used an hourglass network with an encoder–decoder architecture to generate the heatmap for landmark detection of the pelvic images. The landmarks detected were then used to calculate the acetabular index angle and the age of the femoral head. Similarly, Li Q et al. [21] utilized 11,574 orthotopic anterior pelvic X-ray images to train and test a deep-learning model that consists of a modified Mask R-CNN built on FPN and ResNet101. The model detects four key points of sharp angles from pelvic X-ray images and is later used to automate the calculation of the acetabular angle. Mask RCNN architecture has yet another use case where it was implemented by Xu et al. [22] for image segmentation of the pelvis as the first part of a three-stage pipeline. The second stage was built using HRNet to extract the four relevant DDH landmarks. The final stage was a ResNet model providing a binary diagnosis, which was compared with the judgment of three surgeons to evaluate the AI performance on a dataset of 1398 cases. Additionally, Lee et al. [23] used a Mask RCNN model on ultrasound images to segment the shapes formed by the ilium and acetabular head, then assessed two points on the ilium and three points for calculating the α and β, angles using a dataset of 321 ultrasound scans. Zhang et al. [24] built an FR-DDH deep-learning network with baseline of ResNet101 model to extract feature maps and generate potential neighborhood regions to assess the relevant landmarks and calculate the necessary measurements. The study used 10,219 anteroposterior pelvic radiographs from children aged 10 days to 10 years with a mean age of 1.5 years.

Several research in the literature aimed to implement a classification model for diagnosing DDH directly. For instance, in a similar study by [25], authors utilized 13 deep learning CNN pre-trained models to individually perform binary classification of DDH with different input sizes, widths, and depths using the ImageNet dataset fine-tuned on a custom dataset with a size of 354 images. Out of the 13 pre-trained deep-transfer models, DarkNet53 achieved the highest performance with an overall accuracy of 96.3%, equating to 95%, 90.6%, 100% and 94.3% for the F1 score, precision, sensitivity, and specificity, respectively. Park et al. [26] constructed a dataset of 5076 cropped unilateral hip joint images and developed a customized CNN binary classifier to evaluate the abnormality of the extracted unilateral hip joints from AP radiographs.

Similarly, in [27] authors developed an AI-based clinical decision support system in association with non-expert clinical DDH ultrasound staff. In the workflow experiment, the AI-based app exhibited 100% specificity and recommended follow-ups with relatively fewer errors. In [28], authors presented a novel deep learning-based approach to DDH diagnosis by misshapen pelvis landmark detection using local–global feature learning. The technique was name as Pyramid Non-local UNet (PN-UNet). The scheme exhibited good average point-to-point error. In this regard, a self-created dataset comprising 10,000 X-ray images was investigated. Earlier, the same authors presented a similar work on the same dataset but using spatial local correlation mining with CNN. Nonetheless, the later scheme was better in terms of mean absolute error (MAE) [29].

Such types of automated and AI-based studies have been promising in contrast to the general practitioners’ assessments and surveillance which requires mass screening which is laborious as well as erroneous [30].

Likewise, there are several applications of numerical and deep-learning approaches to solve problems in interdisciplinary areas such as physics [31], hyperphysical [32] and healthcare sectors [33,34,35].

#### 1.1.3. Research Gap

Several deep-learning approaches have been utilized to automate DDH diagnosis from medical imaging. Some researchers developed tools to help professionals diagnose DDH by calculating the related clinical measurement, while others aimed to fully automate the process and offer direct binary classification. Nonetheless, a recurrent theme in the literature is primarily observed in the diversity of bone morphology in the imagery dataset due to the large age range in contrast to other factors such as gender and ethnicity. This could be remedied by restricting the ages of the included instances to make it simpler for the deep-learning models to recognize patterns, especially as the available datasets in this scope are of a small size. The lack of practical implementation of the research findings is another identified drawback in the earlier studies. Many have suggested developing applications for portable devices to better aid clinicians in various healthcare domains. However, there have been no advances in that area yet.

Only a few application or software tools are available. These are usually owned by a facility or practice and not available for public use by the physicians. Table 1 presents a comparative analysis of various state-of-the-art methods in landmark detection.

The rest of the paper is organized as: Section 2 presents the materials & methods. Section 3 is dedicated to experimental results. Section 4 provides discussion on the results, while Section 5 concludes the paper.

## 2. Materials and Methods

The dataset used in detecting developmental dysplasia of the hip (DDH) was obtained by the authors of [25] from King Abdullah University Hospital, Jordan University of Science and Technology in Jordan. The dataset included 354 radiograph images of the pelvic area in infants with a mean age of 4.5 ± 0.83, maximizing at 7 months. The dataset format sourced from the main authors is divided into two labels including 120 instances for DDH classified cases, and 234 images of normal condition cases. Nonetheless, the divergence of the use case in this paper calls for additional data annotation measures of the major landmarks in the pelvis guided by experienced radiologists.

### 2.1. Data Annotation

**Figure 2:**
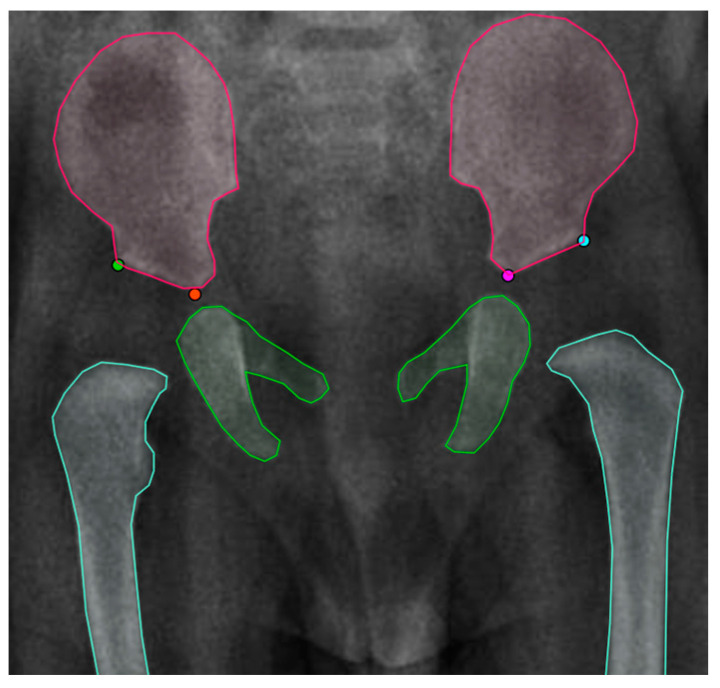
Instance Segmentation and Key point labeling (Adapted from [7]).

The tool CVAT [36] was utilized to annotate the X-ray images. CVAT is an open-source video and image annotation tool. It offers various annotation features for object detection, semantic segmentation, and classification. First, the four landmarks that lie on the Hilgenreiner line and the lateral aspect of the acetabulum roof that passes through the acetabular sourcil’s medial edge were annotated as one object. Each point was labeled according to its position in the X-ray image (left up, left down, right up, right down) indicating the left and right side of the acetabulum superolateral margin and tri-radiate cartilage center. Additionally, the X-ray images were labeled for image segmentation in polygons. The ilium is the upper bone, the pubis, the ischium is the middle bone, and lastly, the femur is the lower bone using the polygon tool. Figure 2 shows the instance segmentation and keypoint labeling.

### 2.2. Statistical Analysis of the Dataset

Given the four pelvis landmarks, a measurement of the acetabular index angle can be calculated using the following formulas:

(1) $$\vec{H}=(\mathrm{RD},\mathrm{LD}),$$

(2) $$\vec{R}=(\mathrm{RU},\mathrm{RD}),$$

(3) $$\vec{L}=(\mathrm{RU},\mathrm{LD}),$$

(4) $$\theta_{M}=\tan^{-1}\frac{\vec{H}.\vec{M}}{det(\vec{H},\vec{M})}$$

where $\vec{H}$ represents the Hilgenreiner line, $\vec{R}$ and $\vec{L}$ represent the side of the line passing through the lateral aspect of the acetabulum to the acetabular sourcil’s medial edge in the respective side, where  M∈{R,L}. Each of the landmarks (RU, RD, LU, LD) represent the co-ordinates of the right side lateral acetabular edge, right-side triradiate cartilage, left-side lateral acetabular edge and left-side triradiate cartilage.

Statistical analysis of the acetabular index angle measurement was conducted on all images within the dataset. The analysis utilizes five number summaries for each class: median, minimum (Min), first quartile (Q1), third quartile (Q3), and maximum (Max). Table 2 enlists the five number summary of acetabula index angle classes in the dataset.

**Figure 3:**
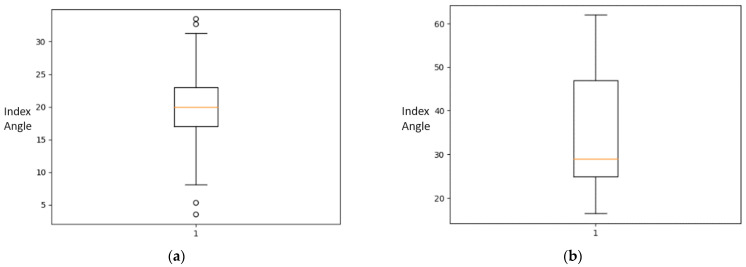
(**a**) Box plot of ACI for Normal infants; (**b**) Box plot of ACI in DDH patients.

Statistical analysis tools can show the presence of outliers in the data. The findings show a mean of 20.0° for acetabular index angle measurement in normal patients. The cases afflicted with DDH however obtained a mean of 30°. The box plot for Normal cases and the DDH cases are shown in Figure 3.

### 2.3. Operationl Framework

Landmark detection is the most critical step for assessing the acetabular index angle in X-ray images and diagnosing DDH afterwards. This research proposes a software tool to fully automate the process of identifying the relevant landmarks in pelvic anatomy and eliminate the need for human intervention.

Nonetheless, choosing the best strategy for a particular task poses a significant challenge that calls for extensive investigation of the most optimized technique to deploy. This project approaches this problem by utilizing the concepts of convolutional neural networks (CNNs). The rationale for this decision is that a CNN is purportedly the most convenient technique to handle computer vision tasks with imagery data as presented in the literature [37,38,39]. CNN is a standardized type of feed-forward neural network that discovers feature space inherently by optimizing the filters. Moreover, the famous vanishing and exploding gradients that are mainly observed during backpropagation-based neural networks are adequately prevented by utilizing the standardized weights over limited connections [40,41].

### 2.4. Experemintal Setup

For developing the pipelined landmarks detection model, the Python programming language was used. The model was trained using Pytorch and Detectron2 frameworks on a Windows workstation equipped with an AMD Ryzen 7 5800X CPU and a 12 GB Nvidia GeForce 3060 GPU. The dataset was randomly split into 70% for the training, 10% for the validation, and 20% for testing.

## 3. Results

In this work, two main approaches were implemented. Namely the baseline landmark detection, and the pipeline-based approach that consists of stacked-up instance segmentation and landmark detection models.

### 3.1. Baseline Landmarks Detection

Landmarks detection describes locating the spatial places and points that are prominent in an image. For assessing the acetabular index angle (AcI), four landmarks within the pelvic X-ray image must be detected: the left and right tri-radiate cartilage center, in addition to the left and right acetabulum superolateral margin. This work utilizes two keypoint detection network architectures and a ResNet50-based model that was fine-tuned to the dataset to produce a final layer with eight outputs. Moreover, Keypoint RCNN architecture was implemented in Detectron2, a Python-based platform for several computer vision tasks. It is a deep neural network used for detecting and localizing human body keypoints based on the Mask R-CNN network architecture proposed by the Facebook research team [42]. Minor changes to the architecture included the addition of a new keypoint head consisting of eight 3 × 3 512-d convolutional layers, followed by a deconvolution layer and 2× bilinear upscaling, producing an output resolution of 56 × 56. This network was adopted and trained with optimal hyperparameters.

### 3.2. Pipeline Landmark Detection

The recognition of anatomical landmarks is made more difficult by the temporal variability and clinical deformity in the medical imaging of infants. Thus, we have attempted to make it smoother for a neural network model to detect edges and locate the landmarks’ positions in a precise manner. Driven by the effectiveness of image segmentation tools to aid medical imaging by providing a more thorough analysis of anatomical data, a pipeline technique was proposed to boost the performance of the landmark detection network. The process to design a pipelined keypoint detection model included constructing a different dataset where all images are already segmented. This dataset was directly exported from CVAT with COCO 1.0 annotation format, then processed using the Detectron2 Visualizer class to generate a customized segment.

#### 3.2.1. First Stage: Instance Segmentation

Image segmentation refers to the task of dividing an image into segments where each pixel in the image is assigned to an object. There are various types of image segmentation tasks including instance segmentation, panoptic segmentation, and semantic segmentation. The scope of this study is concerned solely with implementing the instance segmentation model, where the main aim is to detect the instance of an object and outline its boundaries along with classifying them and applying binary masking as a preparation measure for the upcoming stage.

The technique utilized for this task is implementing Mask R-CNN, a CNN architecture that extends the findings of its predecessors, namely R-CNN, Fast R-CNN, and faster R-CNN. It introduces significant concepts that were proven efficient in object detection fields, such as region proposal network (RPN) and region of interest pooling (ROI pooling) [36]. The basic architecture of MASK R-CNN consists of a backbone, an RPN, a region of interest alignment layer (RoIAlign), a bounding-box object detection head, and a mask generation head. This work utilizes the official Mask RCNN implementation in Detectron2 for instance segmentation. The optimal parameters used to train this network were set to be identical to the baseline Keypoint RCNN described in Table 3.

#### 3.2.2. Second Stage: Landmark Detection

**Figure 4:**
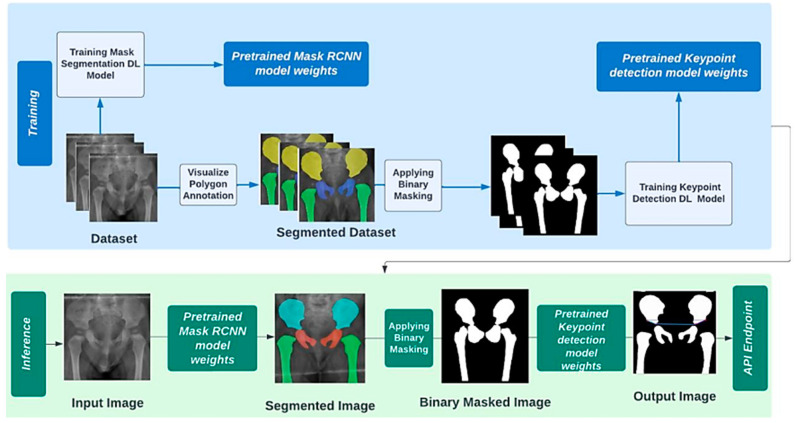
Pipelined segmentation and keypoint detection process.

The network architecture of the second stage is identical to the baseline Keypoint RCNN. The main difference is the data it expects to receive have a format of binary masked images because of visualizing the segmented polygons annotations in the training phase. However, the inference pipeline uses the output generated by passing the instance segmentation model as input to detect the four landmarks. Figure 4 depicts the pipeline segmentation and keypoint detection process.

#### 3.2.3. Performance Metrics

The performance of the model for landmarks detection on the test was evaluated using root mean squared error (RMSE), according to the following equation [43]:

(5) $$RMSE=\sqrt{\sum_{i=1}^{n}\frac{{\left(\hat{y_{i}}-y_{i}\right)}^{2}}{n}}$$

In addition, to present more perceptible results that can be evaluated by medical specialists, metrics such as the acetabular index error rate, and the pixel error were added as well, calculated according to the following equations:

(6) $$Acetabular\;Index\;Angle\;Error=\sum_{i=1}^{n}\frac{\left|\hat{{AcI}_{i}}-{AcI}_{i}\right|}{n}$$

(7) $$Pixel\;Error=\sum_{i=1}^{n}\frac{\sqrt{{\left({\hat{x}}_{i}-x_{i}\right)}^{2}+{\left(\hat{y_{i}}-y_{i}\right)}^{2}}}{n}$$

A comparison between the proposed pipelined technique and the baseline landmarks detection models was carried out as part of the experiments. Additionally, based on the generated acetabular index (AcI) angle measurement in comparison to the ground truth labels obtained from the original dataset, a diagnosis of DDH was made. The RMSE and ACI error rate values for the test dataset before and after prior segmentation are shown in Table 4 correspondingly.

As observed, applying segmentation with binary masking on the Keypoint RCNN model yielded the best results yet in the entirety of this study, surpassing the performance of both ResNet50 based network approaches and the baseline Keypoint RCNN model. Table 5 and Table 6 show the detailed results of AcI error and pixel error achieved by the enhanced Keypoint RCNN detection model.

**Figure 5:**
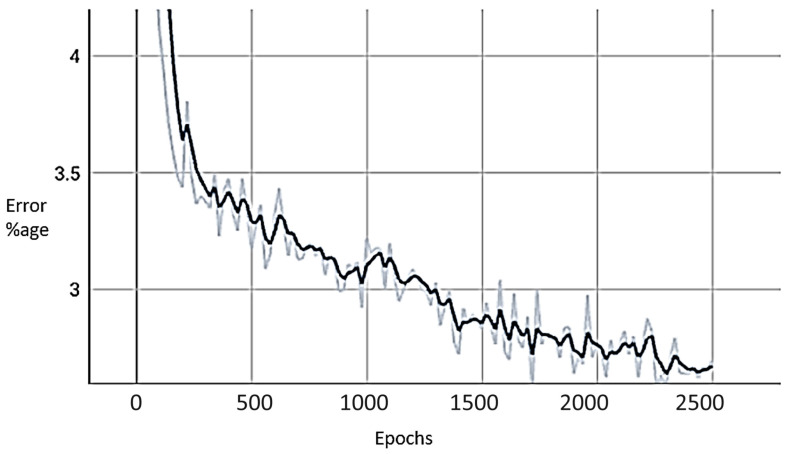
Training and validation loss in pipelined Keypoint RCNN.

Tracking the loss in the model training process is crucial in building deep-learning models. It ensures that the training is going in the right direction. This was positively verified in training the Pipelined Keypoint RCNN model as shown in Figure 5.

## 4. Discussion

Although X-ray imaging is regarded as a reliable tool for DDH diagnosis, a challenge can be presented in relation to the age of screening. The femoral head nuclei start to appear between 4 to 7 months, giving the X-ray imaging screened at that age more ambiguous bone definition, which consequently affect the judgment of the radiologists diagnosing the presented case [44]. Nonetheless, the error rate produced from the AI diagnosis is noticeably low in this study as it achieved a pixel error in landmarks detection of 2.862 ± 2.392 and AcI measurement error of 2.402 ± 1.963°. The highest performance achieved in the literature for landmarks detection used a random forest regression voting model with a point-to-point error of 2.72 mm. For the AcI calculation, the multi-task hourglass network achieved the lowest error in the prior work with an ACI angle error of 2.776 ± 2.381° [20]. The slight performance boost in this study, despite the small size of the dataset, could be attributed to adopting a multistage pipeline where detecting the landmarks is preceded by segmenting the images. Moreover, with a dataset of similar bone morphology, AI models are more inclined to recognize a pattern with less error rate. Nonetheless, it can be observed that the performance metrics of the prior works are of high divergence. Thus, standardized evaluation methods are highly capable of comparing the performance of works within the same scope.

This study has a few limitations including the small-sized dataset and the low quality of images in addition to ensuring the reliability of labeling in the utilized open-sourced dataset by experienced radiologists. Moreover, the lack of patient clinical data in each case such as age, gender, and ethnicity can affect the final judgment of DDH affliction, which currently is solely dependent on the anatomical landmarks on the medical image. Additionally, more radiographic measurement can be taken into consideration besides acetabular index angle, namely the sharp angle and lateral center-edge angle for supportive assessment of the case.

To address these issues, future work can extend the findings of this study by including more radiographical measurements to cover more pelvis related diseases in conjunction with transfer learning [45]. Additionally, utilizing online deployed models to continuously feed the model with new data that are validated by the medical professionals of the system will improve the long-term performance of the automatic DDH diagnosis. Moreover, the authors intend to deploy the deep-learning model in a working mobile application to be released soon and adopted by local hospitals in Saudi Arabia.

## 5. Conclusions

This study introduces the pipeline landmarks detection technique, a novel method for detecting DDH, which involves applying binary masked image segmentation before passing the data to the landmark’s detection network. The landmarks detection model based on the Detectron2 implementation was trained on the considered diverse open dataset, achieving 2.402 ± 1.963° for the acetabular angle measurement relative to the ground truth annotation and pixel error of 2.862 ± 2.392 for the landmarks. These values indicate an error range that is acceptable in medical and professional fields. The contributions this work has made included:

- Fully annotating an open-source dataset [7] for instance segmentation and landmarks detection, which was published solely for binary classification task.
- Building an efficient deep-learning landmarks detection model with error margin of 2.402 ± 1.963° for the acetabular index angle measurement.

Overall, the proposed methodology in this work can serve as a useful reference and be used broadly to a variety of anatomical landmark identification problems on a larger scale and more reliable dataset with high potential of boosting the current performance. In the future, we intend to investigate further deep-learning paradigms such as transfer learning etc. in conjunction with image-processing approaches to improve the performance and make it robust against potential changes in the dataset type.

## Figures and Tables

**Table 1:** Comparative analysis of different state-of-the-art methods for landmark detection.

Ref.	Dataset Size	Methods	Performance	Limitation
[18]	400	Random Forest Regression Voting Model	Point-to-point error: 2.72 mm	Lack of implementation details for reproducibility.
[20]	9369	Multi-Task Hourglass Network with Encoder-Decoder	Landmarks Pixel Error: 4.67 ± 5.3 ACI Angle Error: 2.776 ± 2.381°	Extreme diversity of bone morphology in these X-ray images due to large age range
[21]	11,574	Mask R-CNN built on FPN and ResNet101	AI measurement: 40.36 ± 4.15° Surgeons’ measurement: 39.59 ± 6.87°	Solely assessing sharp angle
[22]	1398	Mask R-CNN, HRNet, ResNet50 for segmentation, keypoint detection, and classification respectively.	Landmarks Mean Euclidean distance error: 4.653	Lack of integration with clinical practice
[23]	321	Mask R-CNN for segmentation	Base Angles Difference: 1.81°	Solely used Ultrasound scans Lack of implementation details for keypoint detection model
[24]	10,219	FR-DDH network with ResNet-101 for feature map and spatial information extraction	Bland–Altman 95% limits of agreement for AcI: −4.733°	Extreme diversity of bone morphology in X-ray images for wide age range Lack of integration with clinical practice
[28]	10,000	CNN—Pyramid Non-local UNet (PN-UNet)	Average point-to-point error: 9.286 µm	Local dataset with manual annotations

**Table 2:** Five number summary of acetabular index angle classes.

Five Number Summary		
DDH Angle	Minimum	16.442
	Q1	24.869
	Median	28.931
	Q3	47.278
	Maximum	61.932
	IQR	22.409
Normal Angle	Minimum	3.498
	Q1	16.968
	Median	19.947
	Q3	22.976
	Maximum	33.479
	IQR	6.008

**Table 3:** Optimal hyperparameters for Keypoint RCNN.

Model Hyperparameters	
RPN Batch Size	256
Roi Heads Batch Size	128
Base Learning Rate	0.00075
Maximum Iteration	2500
Weight Decay	0.0001
Batch Size	4

**Table 4:** Comparing the performance of the proposed approaches.

Model	RMSE	AcI Error	Pixel Error
Baseline ResNet50	7.108	4.3705 ± 3.565	7.141 ± 6.7355
Pipelined ResNet50	3.408	3.3275 ± 2.94	4.266 ± 3.545
Baseline Keypoint RCNN	2.9	2.955 ± 2.51	3.309 ± 2.410
**Pipelined Keypoint RCNN**	**2.7**	**2.402 ± 1.963**	**2.862 ± 2.392**

**Table 5:** Pixel error of Pipelined Keypoint RCNN.

Pipelined Keypoint RCNN with Binary Mask Segmentation: Pixel Error				
	Mean	Std	Median	Mean ± Std
Landmark RU	2.582	1.908	2.204	2.582 ± 1.908
Landmark RD	3.287	2.947	2.35	3.287 ± 2.947
Landmark LU	3.584	3.107	2.656	3.584 ± 3.107
Landmark LD	1.993	1.607	1.552	1.993 ± 1.607
Avg	2.862	2.392	2.190	2.862 ± 2.392

**Table 6:** ACI error of pipelined Keypoint RCNN.

Pipelined Keypoint RCNN with Binary Mask Segmentation: AcI Measurement Error Rate				
	Mean	Std	Median	Mean ± Std
Left	2.127	1.732	1.996	2.127 ± 1.732
Right	2.676	2.194	2.37	2.676 ± 2.194
Avg	2.402	1.963	2.183	2.402 ± 1.963

## Data Availability

Data is available with the corresponding author and can be provided on reasonable request.
